# Isolation, Molecular, and Histopathological Patterns of a Novel Variant of Infectious Bursal Disease Virus in Chicken Flocks in Egypt

**DOI:** 10.3390/vetsci11020098

**Published:** 2024-02-19

**Authors:** Ahmed H. Salaheldin, Hatem S. Abd El-Hamid, Hany F. Ellakany, Mostafa A. Mohamed, Ahmed R. Elbestawy

**Affiliations:** 1 Department Poultry and Fish Diseases, Faculty of Veterinary Medicine, Alexandria University, Alexandria 21944, Egypt; 2 Department of Bird and Rabbit Diseases, Faculty of Veterinary Medicine, Damanhour University, Damanhour 22511, Egypt; drhatem_deltavet@yahoo.co.uk (H.S.A.E.-H.); ellakany_hany@vetmed.dmu.edu.eg (H.F.E.); 3 Department of Pathology, Faculty of Veterinary Medicine, Menoufia University, Shebeen Elkom 32511, Egypt; mostafaabdelgaber@vet.menofia.edu.eg; 4 Department of Bird and Rabbit Diseases, Faculty of Veterinary Medicine, Menoufia University, Shebeen Elkom 32511, Egypt

**Keywords:** nVarIBDV, vvIBDV, VP2, A2d, chickens, Egypt

## Abstract

**Simple Summary:**

Infectious bursal disease virus (IBDV) is a potent immunosuppressive, persistent pathogen that can thrive in a range of environmental conditions and withstand even potent disinfectants. The common circulating pathotypes of IBDV in Egypt are classical virulent, attenuated, very virulent IBDV, and novel variant (nVarIBDV). The current study describes the incidence of nVarIBDV in 18 Egyptian chicken flocks although they were vaccinated against IBD. Partial sequence analysis of viral protein 2 (VP2) in two obtained isolates identified them as nVarIBDV (genotype A2d) exhibiting 100% identity to SD-2020 and 99.5–98.1% similarity to ZD-2018-1, QZ, GX and SG19 strains. Similarity to USA variant strains was 95.5–92.6%. Moreover, the similarities in the aa of the VP2 hypervariable region of both isolates to vaccine strains were 86–90.4%. Histopathological examination of both the bursa of Fabricius and spleen collected from diseased chickens in flock no. 18 revealed severe atrophy. In conclusion, this study identified the nVarIBDV genotype which is identical to the nVarIBDV circulating in China. Further studies are required to investigate the epidemiological situation of this novel genotype across the country, and to assess various vaccine protections against nVarIBDV. Additionally, it is crucial to incorporate nVarIBDV into the inactivated vaccines administered to breeder chickens prior to egg production to ensure complete protective and specific maternal immunity in their offspring during their first three weeks of life.

**Abstract:**

After an extended period of detecting classical virulent, attenuated, and very virulent IBDV, a novel variant (nVarIBDV) was confirmed in Egypt in this study in 18, IBD vaccinated, chicken flocks aged 19–49 days. Partial sequence of viral protein 2 (VP2) [219 aa, 147–366, resembling 657 bp] of two obtained isolates (nos. 3 and 4) revealed nVarIBDV (genotype A2d) and OR682618 and OR682619 GenBank accession numbers were obtained. Phylogenetic analysis revealed that both nVarIBDV isolates were closely related to nVarIBDV strains (A2d) circulating in China, exhibiting 100% identity to SD-2020 and 99.5–98.1% similarity to ZD-2018-1, QZ, GX and SG19 strains, respectively. Similarity to USA variant strains, belonging to genotypes A2b (9109), A2c (GLS) and A2a (variant E), respectively, was 95.5–92.6%. Also, the VP2 hypervariable region in those two, A2d, isolates revealed greater similarities to Faragher 52/70 (Vaxxitek^®^) at 90.4% and to an Indian strain (Ventri-Plus^®^) and V217 (Xtreme^®^) at 89.7% and 86–88.9% in other vaccines. Histopathological examination of both the bursa of Fabricius and spleen collected from diseased chickens in flock no. 18 revealed severe atrophy. In conclusion, further studies are required to investigate the epidemiological situation of this novel genotype across the country, and to assess various vaccine protections against nVarIBDV. Additionally, vaccination of breeders with inactivated IBD vaccines including this nVarIBDV is essential to obtain specific maternal antibodies in their broilers.

## 1. Introduction

Infectious bursal disease (IBD), also known as Gumboro, is a highly significant viral disease in young chickens with considerable economic implications due to high mortality rates and immunosuppression [1]. Generally, serotypes 1 (pathogenic) and 2 (non-pathogenic) of infectious bursal disease virus (IBDV) have been identified in the literature [2,3,4,5]. Previously, serotype 1 consisted of three primary genetic types. The classical genotype (cIBDV) initially emerged in 1957, and the detection of variant IBDV (varIBDV) followed in 1984 in Gumboro county, Delaware, USA. In 1987, very virulent (vvIBDV) strains appeared in Europe, specifically in the Netherlands and Belgium [6].

Recently, Michel and Jackwood [7] and Jackwood et al. [8] proposed a novel genotyping system platform which utilizes nucleotide sequences from the hypervariable region (HVR) in the viral protein 2 (VP2) of segment A of serotype 1 only and classified IBDV strains into seven genogroups (1–7) without any reference to segment B or serotype 2. Their investigation concluded that there are seven genogroups of infectious Bursal Disease Virus (IBDV) that exist worldwide. Further genogrouping criteria provided greater detail based on segments A (VP2-HVR) and B (VP1) of both serotype 1 and 2. Wang et al. [9] classified IBDV into nine genogroups, combining both early and variant Australian strains into A7, and separated the classically attenuated viruses into the A8 group, away from A1 (classically virulent) and AII for segment A of serotype 2. For segment B, they added only five genogroups, B1-B4 for serotype 1, and BII for serotype 2. Furthermore, Islam et al. [10] identified eight genogroups of segment A (A1–8) in which A1a belonged to classical virulent strains, A1b was assigned to classical attenuated strains, A7 and A8 were the early and variant Australian strains, respectively, and five genogroups of segment B (B1–5) of serotype 1, as well as a single genogroup under serotype 2, were named A0 and B1. Based on some controversial areas such as cIBDV or vvIBDV being derived from attenuated strains, and thus they are included in separate subgroupings to help define reassorting virulent stains, Gao et al. [11] proposed a new classification system. This new classification system includes subgroups of segment A (1–9), in which A7 were the early Australian strains, A8 were the Australian variants, and A9 were the attenuated viruses, to avoid any confusion to the genogrouping previously reported by Islam et al. [10].

IBD was initially reported in Egypt during the early seventies by El-Sergany et al. [12], who detected the viral impact via histopathological examination. Subsequently, Ayoub and Malek [13] successfully isolated and identified classical IBDV from diseased broiler chicken flocks aged 3–5 weeks old. Meanwhile, vvIBDV was first observed in vaccinated chicken flocks in 1989 by El-Batrawi [14], and it remained the most common IBDV genotype in the field until 2022 [15,16,17,18,19,20,21,22,23,24,25]. The following sources have investigated the detection of variant IBDV in Egypt: Amer and Nassif [26] attempted to detect the virus in three pooled proventricular homogenates obtained from three broiler chicken flocks aged 15 to 30 days old. The identity of the isolates as the Del/E variant strain of IBDV was determined through serological diagnosis using the agar gel precipitation test (AGPT) with reference antibodies against IBDV, along with the conventional reverse transcriptase polymerase chain reaction (cRT-PCR) and restriction fragment length polymorphism (RFLP) test on PCR products using MboI and BstOI restriction enzymes. The lack of sequence analysis hindered the accurate identification of these variant strains. However, recently Legnardi et al. [27] have detected nVarIBDV (A2dB1b) from broiler chickens in Egypt during 2022–2023.

Classical virulent infectious bursal disease virus (cIBDV) strains lead to damage to the bursal and lymphoid tissues, resulting in mortality rates of 10–20%. On the other hand, the very virulent infectious bursal disease virus (vvIBDV) strains cause significantly higher mortality rates of 20–30% and 60–70%, respectively, in susceptible broiler chickens aged 3–6 weeks and layer pullets aged 3–10 weeks [6]. Infection with the IBD variant in young chickens within the first three weeks of life results in more severe illness, even in the absence of mortality rates, due to the destruction of precursor antibody-producing cells in the bursa of Fabricius, notably B-lymphocytes, causing bursal atrophy and permanent immunosuppression. This leads to a reduced ability to eliminate the virus and weakened antibody response to vaccines. Post-vaccination adverse effects, heightened vulnerability to accompanying or subsequent ailments, and diverse consequences of infections including Newcastle disease (ND), low pathogenicity avian influenza H9N2 (LPAI-H9N2), adenovirus or inclusion body hepatitis, chicken infectious anemia virus (CIAV), infectious bronchitis virus (IBV), *Escherichia coli*, Mycoplasma, necrotic enteritis or gangrenous dermatitis and coccidiosis have been observed [28,29,30,31].

Since the start of 2023, early disease complex challenges have been identified in all types of chickens, notably broilers. The complex manifests at 15 days old, consistently accompanied by atrophy of the bursa of Fabricius. This highlights the concerns of premature immunosuppression due to the variant IBDV infection in Egypt. Therefore, this study aimed to isolate and identify variant IBDV in diseased chicken flocks in Egypt. Sequence and phylogenetic analysis were performed on some obtained IBDV strains, targeting VP2.

## 2. Materials and Methods

### 2.1. Ethical Approve

The examination of chickens in this study adhered to the guidelines on research ethics and was approved by the Institutional Animal Care and Use Committee (IACUC) at the Faculty of Veterinary Medicine, Alexandria University. Every effort was made to minimize the suffering of birds. The Ethical Approve Code was ALEXU/VetMed-2023/025. The owners of the sampled farms gave their official consent prior to the examination.

### 2.2. Examination and Sampling of Chickens

Fifteen pooled bursae of Fabricius and 3 pooled bursae and spleen tissues from chickens in 18 diseased flocks were obtained. These flocks consisted of 15 broiler flocks and 3 indigenous Balady flocks, aged between 19 and 49 days old, from 4 Egyptian governorates, namely Beheira (*n* = 10), Alexandria (*n* = 6) and New Valley (*n* = 2). Table 1 explains the history including IBD vaccination programs using various live and recombinant vaccines. Three to five pooled hemorrhagic and/or swollen edematous bursa were collected from each flock under hygienic conditions and packaged in properly labelled plastic bags. The samples were then combined and homogenized in PBS containing 1000 IU/mL penicillin G and streptomycin each, before being centrifuged at 3000× *g* rpm for 10 min. The supernatants were transferred into new sterile Eppendorf tubes and stored at −80 °C for further investigations [32].

### 2.3. Viral Isolation and Propagation in Specific Pathogen-Free Embryonated Chicken Eggs (SPF-ECE)

Tissue homogenates were prepared and then introduced into 90 fertile SPF-ECE eggs, aged 12 days, via chorioallantoic membrane (CAM) using a dose of 0.2 mL per egg. These eggs were subsequently incubated at 37 °C and monitored for 5 days after inoculation by candling. Mortalities were documented, and subsequently, both chorioallantoic membranes (CAMs) and 2 mL of allantoic fluid (AF) were collected from each egg. The samples were then combined, homogenized in sterile PBS, and the resulting supernatants were placed into new sterile Eppendorf tubes and stored at −80℃ for further investigation [32].

### 2.4. Real Time Reverse Transcriptase Polymerase Chain Reaction (rRT-PCR)

All CAM and AF homogenates were analyzed for IBDV infection using vvIBDV and non-vvIBDV specific primers along with two TaqMan probes [33]. The study also included assays for detection of other viral co-infections such as Newcastle Disease [34], avian influenza [35], infectious bronchitis [36], adenovirus [37], reovirus [38], and chicken infectious anemia virus [39]; they were detected via rRT-PCR using specific primers and cycling conditions. To extract viral RNA, 200 μL of bursal homogenate supernatant from each pooled sample was mixed with 1 mL of GENEzol™ Reagent (Geneaid, New Taipei City, Taiwan). The manufacturer’s protocol was followed, using the One-step RT-PCR master mix kit from IDEXX (Hoofddorp, Netherlands).

### 2.5. VP2 Sequence Analysis and Phylogenetic Tree

Two Egyptian Infectious bursal disease virus (IBDV) isolates, numbers 3 and 4, underwent partial sequencing of 219 amino acids (147–366, equivalent to 657 base pairs) that encompassed the hypervariable region of VP2 (VP2-HVR, amino acids 210–350) using the Kylt^®^ RNA/DNA Purification Kit (SAN Group Biotech Gmb, HHöltinghausen, Germany) following the procedure of the manufacturer. The purified PCR products underwent direct sequencing using the ABI PRISM-BigDye^TM^ Terminators v3.1 Cycle Sequencing Kit (Applied Biosystems, Foster City, CA, USA) and the ABI PRISM 3130 genetic analyzer (Applied Biosystems). The sequences underwent editing utilizing SeqScape-Software Version 2.5 (Applied Biosystems). The assembly of consensus sequences and alignment trimming were subsequently performed through employment of the Laser gene DNASTAR program suite (DNASTAR Inc., Madison, WI, USA). The Clustal V technique was used for this process [40]. To locate other related IBDV nucleotide sequences, the nucleotide sequences used in the study underwent blasting through the National Center for Biotechnology Information (NCBI) website (http://www.ncbi.nlm.nih.gov/), accessed on 30 November 2023. Phylogenetic analysis comprised of two nVarIBDV isolates and 91 IBDV sequences downloaded from GenBank, which included 42 isolates from USA variant A2a,b,c, 49 isolates from novel variants (China) A2d, and 53 isolates from other genotypes A1, A3–9 and SIIA. In addition, an alignment comparison was made between the two nVarIBDV and 25 strains (including novel, US variants, classical virulent and attenuated, and vvIBDV) containing 219 amino acids (147–366), including the VP2-HVR (aa 210–350). The nucleotide sequences were uploaded and analyzed through BioEdit (Ibis Bioscience, Carlsbad, CA, USA) and Geneious Prime v 1.0.2022 software packages for molecular evolutionary genetics. Subsequently, the Interactive Tree of Life (iTOL) v5 program was employed to generate phylogenetic trees and annotation, following the methodology proposed by [41].

### 2.6. Histopathological Examination of Field Samples

Bursa of Fabricius and spleen tissue samples were collected from diseased chickens (flock no. 18) and fixed in 10% neutral buffered formalin. Tissue samples were processed routinely and embedded in paraffin wax. Sections were cut with a thickness of 5 μm and stained with hematoxylin and eosin for microscopical evaluation [42]. Bursa lesion scores were determined as follows: score 1 = 1–25%, 2 = 26–50%, 3 = 51–75% and 4 = 76–100% of follicles showing cellular depletion [43].

## 3. Results

### 3.1. Isolation and Identification of nVarIBDV

**Figure 1:**
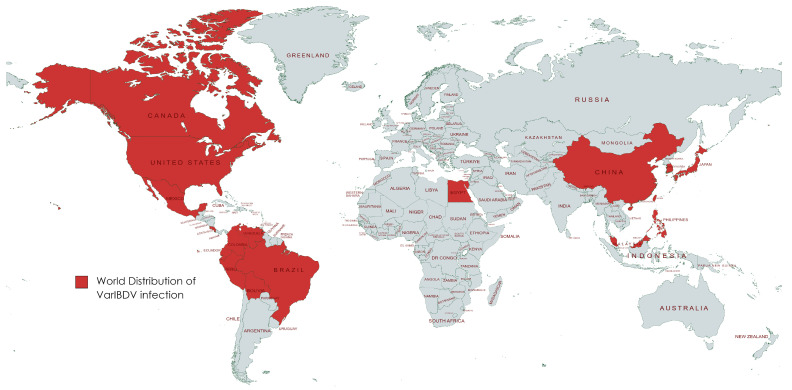
World distribution of VarIBDV infection.

**Figure 2:**
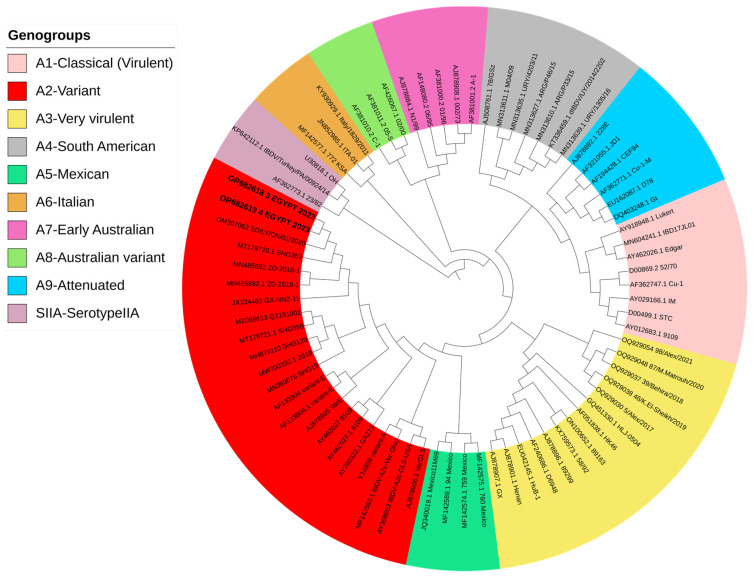
Phylogenetic analysis (polar format) of two nVarIBDV isolates (Accession no. OR682618 and OR682619) isolated in 2023 (bold letters) compared to other IBDV isolates from different genogroups. Interactive Tree of Life (iTOL) v5 program was used to produce phylogenetic tree. Bootstrapping using UFBoot2 method was applied.

**Figure 3:**
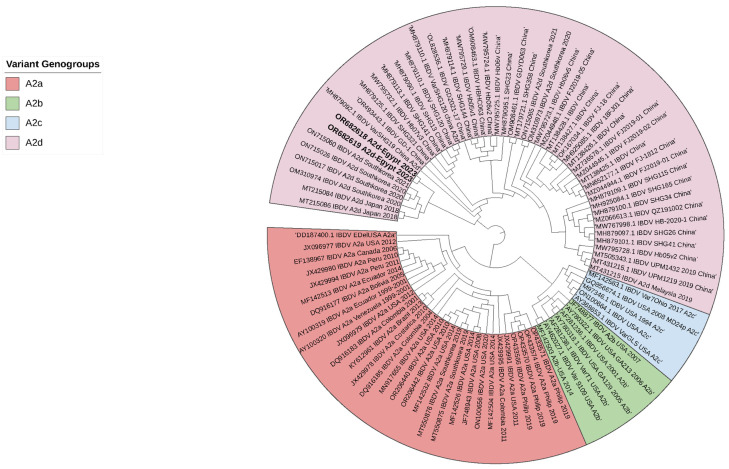
Phylogenetic analysis (polar format) of two nVarIBDV isolates (Accession no. OR682618 and OR682619) isolated in 2023 (bold letters) compared to other recorded variant IBDV isolates in America, China, South Korea and Japan. Interactive Tree of Life (iTOL) v5 program was used to produce phylogenetic tree. Bootstrapping using UFBoot2 method was applied.

All examined chicken flocks exhibited respiratory and digestive disorders, along with postmortem lesions including bursa of Fabricius atrophy, enteritis, pneumonia, tracheitis, fibrinous airsacculitis, perihepatitis and pericarditis, exemplifying complicated chronic respiratory disease (CCRD), with low mortality rates ranging from 0.3 to 2% within five days of the disease course. The assessment of 18 vaccinated chicken flocks aged between 19 and 49 days for IBDV infection using rRT-PCR showed non-vvIBDV in all bursal samples. Moreover, rRT-PCR for other viral diseases disclosed exclusively low pathogenic avian influenza subtype H9N2 (LPAI-H9N2) in all broiler samples (*n* = 15), alongside the suspected CCRD following postmortem examination. Isolation of IBDV in 12-day-old SPF-ECE through inoculation of tissue homogenates via chorioallantoic membrane caused embryonic mortalities within 5 days post-inoculation. The dead embryos exhibited lesions of dwarfing accompanied by congestion and hemorrhages. Real-time RT-PCR detected a non-very virulent infectious bursal disease virus in all samples. Partial sequence analysis of VP2 (219 amino acids, residues 147–366, resembling 657 base pairs) in two isolates (No. 3 and 4) confirmed the detection of the variant infectious bursal disease virus (genotype A2d). Both isolate sequences were submitted to GenBank with the accession numbers OR682618 and OR682619, respectively. The distribution of variant IBDV infections worldwide is indicated in Figure 1. The phylogenetic analysis of A2d classified isolates with other nine genotypes of segment B (A genotype) is indicated in Figure 2. Both isolates were closely related to nVarIBDV strains (A2d) circulating in China, as they shared 100% identity with IBD/SD/LY/CN/01/2020 (accession no. OM307063), were 99.5% similar to ZD-2018-1 (accession no. MN485882) and had 98.1–98.6% similarity to the QZ, GX, and SG19 strains, respectively. Also, both obtained nVarIBDV isolates showed 93.6–97.2% similarity to the USA variant strains A2b (9109), A2c (GLS), and A2a (variant E), as demonstrated in Figure 3.

**Figure 4:**
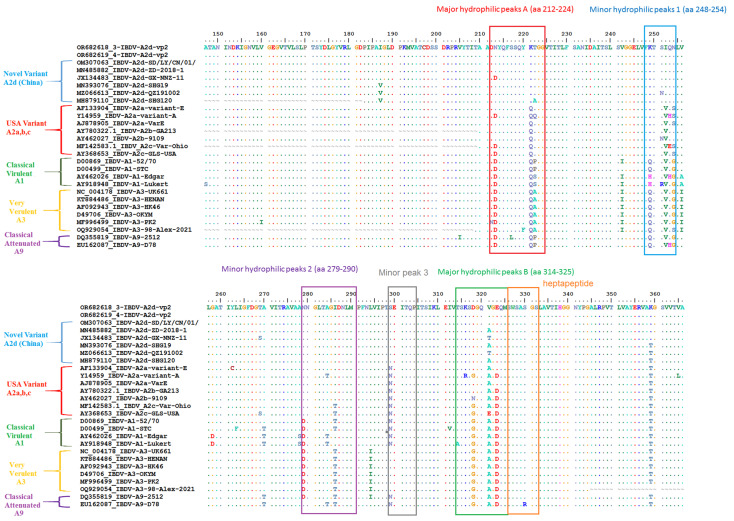
Amino acid differences in VP2 (aa 147–366) of the two nVarIBDV isolates (Accession no. OR682618 and OR682619) isolated in 2023 and other nVarIBDV, variant IBDV (USA), cIBDV (attenuated) and vvIBDV isolates.

**Figure 5:**
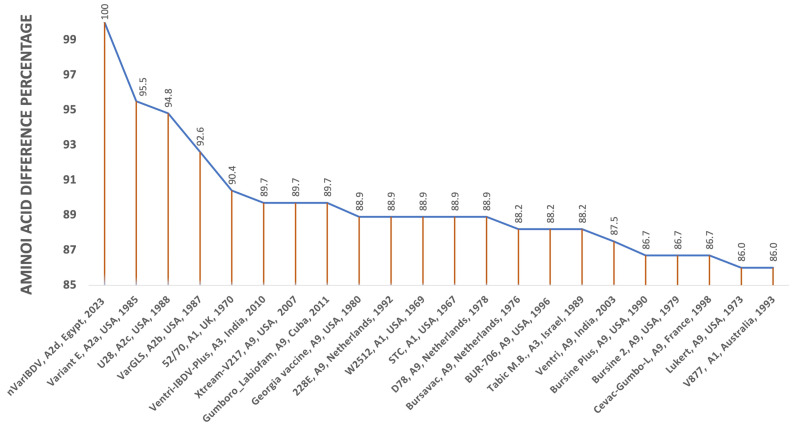
Similarity percent in HVR of VP2 (aa 210–350) of two nVarIBDV, A2d, isolates (Accession no. OR682618 and OR682619) isolated in 2023 and other 21 IBDV strains, three variant A2a, b, c and 18 vaccinal strains (4 of A1, 2 of A3, 12 of A9).

The four loop structures within the hypervariable region (HVR) of VP2, labelled PBC (204–236 aa), P_DE_ (240–265 aa), P_FG_ (270–293 aa) and P_HI_ (305–337 aa), are entirely the same (100%) in our two Egyptian isolates and the nVarIBDV strains (A2d) circulating in China, SD-2020. They are also 99.5% similar to a Chinese strain, ZD-2018-1, with a single aa change (A321V) in the P_HI_ loop. However, both nVarIBDV isolates have multiple aa differences compared to other Chinese and USA variants, cIBDV and vvIBDV examined isolates, as depicted in Figure 4. Within the aa 210–350 sequence of VP2-HVR, the comparison of the two nVarIBDV Egyptian isolates obtained here to various vaccine strains revealed a higher percentage of similarity to Faragher 52/70 (A9) (Vaxxitek^®^) with 90.4%, followed by 89.7% similarity to an Indian strain (Ventri-Plus^®^), V217 (Xtreme^®^) and 88.9% to 228E, W2512, STC and D78 strains and the lowest identity was 86% to Lukert and V877 strains (Figure 5).

### 3.2. Histopathological Examination

**Figure 6:**
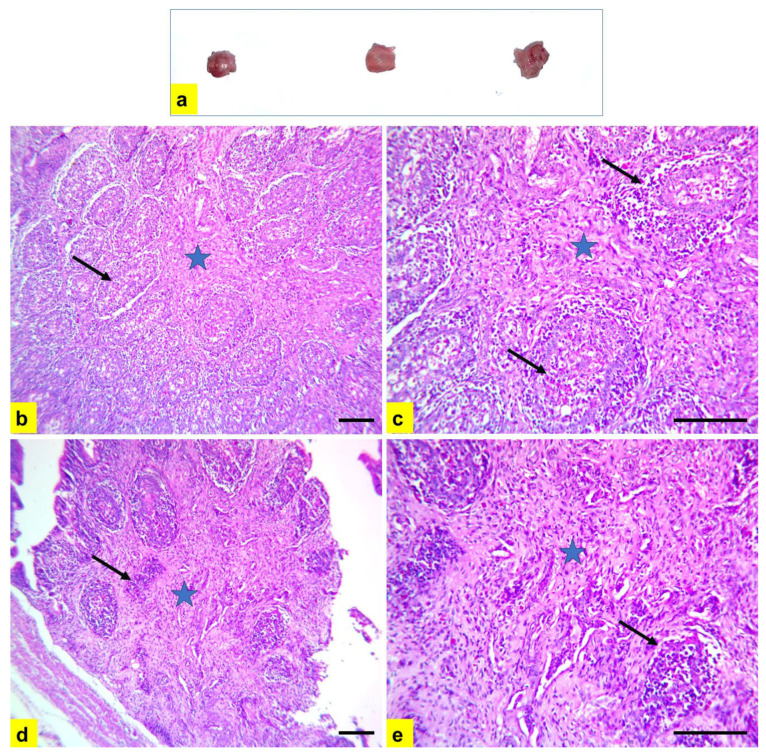
Postmortem, and histopathological lesions in bursa of Fabricius samples from chickens in flock no. 18. (**a**) Severe atrophy of bursa of Fabricius. (**b**–**e**) Severe diffuse depletion and necrosis of lymphoid follicles (black arrow). Severe connective tissue proliferation in-between lymphoid follicles leading to pressure atrophy (blue star). H and E stain; scale bar 100 μm. (**b**,**d**) ×200. (**c**,**e**) ×400.

**Figure 7:**
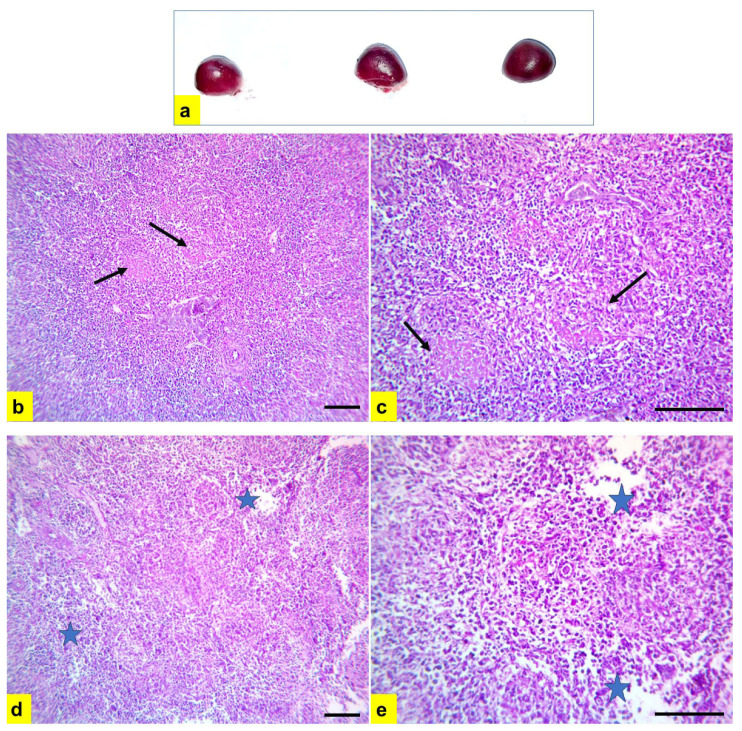
Postmortem, and histopathological lesions in spleen samples from chickens in flock no. 18. (**a**) Severe atrophy of spleen. (**b**,**c**) Severe depletion and rarefication of lymphoid follicles (black arrow). (**d**,**e**) Severe multifocal to diffuse areas of splenic necrosis (blue star). H and E stain; scale bar 100 μm. (**b**,**d**) ×200. (**c**,**e**) ×400.

Severe atrophy of the bursa of Fabricius and spleen were detected during gross examination of diseased chickens (Figure 6a and Figure 7a). Microscopic examination of bursal tissue samples (Figure 6b–e) showed severe diffuse depletion and necrosis of lymphoid follicles with the highest lesion score of 4, indicating severe connective tissue proliferation in-between lymphoid follicles, leading to pressure atrophy. In addition, spleen samples showed severe depletion and rarefication of lymphoid follicles (Figure 7b,c) with severe multifocal to the diffuse areas of splenic necrosis (Figure 7d,e).

## 4. Discussion

The permanent immunosuppressive form of IBDV is caused by variant or low-to-moderate pathogenic strains. These strains have been described mainly in the United States, including A2aB1 (Delaware A, D, E, and G), A2bB1 (9109 and T1) and A2cB1 (GLS). Moreover, novel variant strains of IBDV have recently been detected in China, such as A2dB1 (SD-2020). ZD-2018-1, SHG19, SHG120, Gx-NNZ-11, and QZ191002 were found to have limited clinical impact, low mortality rates and resulted in marked bursal atrophy with little or no immune response [7,44,45]. On the other hand, classical viruses such as the IM strain induced a severe bursal inflammatory response [46,47]. It has been widely reported that these variant strains are only partially resistant to neutralizing antibodies produced by classical or standard strains [32,46,48,49]. In addition, differences in the pathogenicity of IBDV have been observed between different chicken breeds, with generally lighter laying or indigenous breeds (such as Balady and Sasso) being more susceptible compared to heavier broiler breeds [50].

The chickens examined in this study showed respiratory and digestive lesions, atrophy of the bursa of Fabricius and a relatively high mortality rate (up to 2% within 5 days). With this in mind, we sought to investigate the presence of variant infectious bursal disease virus among other viral infections. Subsequently, non-vvIBDV was detected in all bursal samples by rRT-PCR. Partial sequence analysis of VP2 in two isolates (nos. 3 and 4) confirmed nVarIBDV (genotype A2d). Histopathological examination of the bursa of Fabricius and spleen tissues collected from diseased chickens during nVarIBD infection revealed severe diffuse depletion and necrosis of lymphoid follicles in both organs. Similar results were previously obtained by Fan et al. [51], and Lian et al. [52] using novel variant strains, Hb06v and SHG19, respectively. The entry of this nVarIBDV (genotype A2d) into Egypt is difficult to explain, but China has become the epicenter of nVarIBDV spreading to other parts of the world, with a strong correlation between virus transmission patterns and the flow of commercial trade in live poultry and products [53] or migratory birds that could be carriers or spreaders of IBDV [54]. Further studies should be undertaken to provide important insights into the origin, evolution and transmission of nVarIBDV in order to assist in the development of control strategies.

This new threat, novel variant IBDV (nVarIBDV), has emerged in Egyptian chicken flocks during 2023 [27]. nVarIBDV causes severe damage to the central immune organ, the bursa of Fabricius, resulting in permanent immunosuppression in affected chickens, and resulting in reduced production performance (subclinical IBD). Subclinical infection, direct bursal atrophy induced by VarIBDV strains, and also the absence of specific or complete immunity in birds due to the use of the available classical (live and inactivated), recombinant or immunocomplex vaccines do not allow for an accurate preliminary diagnosis, favoring the spread, circulation and evolutionary antigenic divergence of VarIBDV, which may frustrate control measures. It has been reported that nVarIBDV has the ability to inhibit immune responses to vaccines against highly pathogenic avian influenza [46] and Newcastle disease [51,55], which are two of the most serious infectious diseases threatening poultry production. In one particular study, the body weight of broilers infected with nVarIBDV at 42 days old was reduced by approximately 16% compared to the control, resulting in significant economic losses [56]. Furthermore, co-infection of nVarIBDV (ZD-2018-1) with other pathogens, such as FAdV-4-HB1501, increased the pathogenicity of FAdV-4 in SPF chickens, resulting in extensive immunosuppression [31]. The detection of LPAI-H9N2 in all broiler samples in this study highlights the importance of paying attention to mixed infections with nVarIBDV and LPAI-H9N2 in Egypt. This confirms the previous reports indicating that IBD may predispose and exacerbate the magnitude of LPAI-H9N2 in the field [57]. Motamed et al. [58] found that previous infection with IBDV may promote replication and alter the tissue tropism of LPAI-H9N2, thereby prolonging its shedding period from the trachea and cloaca in broiler chickens, which may lead to higher mortality rates. However, more a severe disease condition has been observed with simultaneous natural co-infection with Newcastle disease, LPAI-H9N2 and infectious bronchitis [24,59].

This study reports the detection of nVarIBDV in Egypt by VP2 sequencing, using 2 isolates with GenBank accession numbers of OR682618 and OR682619. Previously, researchers in Egypt have detected antigenically variant IBDV isolates, including the Del/E strain and other untypeable variants, by capturing IBDV antigens using VP2 monoclonal antibody coated plates [60,61]. Hussein et al. [62] studied 46 broiler flocks vaccinated with classical infectious bursal disease (IBD) vaccines, aged 2–4 weeks, collected from four Egyptian governorates and suffering from proventriculitis associated with runting-stunting syndrome. The IBDV variant was detected in the bursa of Fabricius by ELISA and electron microscopy. While these have been the only methods available to date to identify and classify IBDV, it is not possible to officially verify variant IBDV strains by these methods alone. Sequence analysis of VP2 and/or VP1 is essential. In relation to early proventriculitis and its association with infectious bursal disease virus (IBDV), Elkady et al. [63] showed that the vvIBDV FAY97 strain caused a marked proventriculitis after experimental infection of 1-day-old broiler chicks and detected vvIBDV in proventricular samples from commercial broiler chicks at 3–4 weeks of age and in homogenized chorioallantoic membranes from inoculated SPF-ECE using the immunofluorescent antibody (IFA) technique and DOT-ELISA, respectively.

For a considerable period of time, vvIBDV has been the predominant circulating genotype of IBDV in Egypt, exhibiting both antigenically typical and modified patterns (with a single amino acid mutation at position 321 of VP2 situated within the loop PHI, 305–337 aa) during the last 15 years in Egypt. This has led to the emergence of Gumboro disease and acute mortality in susceptible chickens within 5–8 days of infection [24,64]. Genetic reassortment was observed in an isolate from Domiatta, Egypt in 2015 (No. 160019). VP2 was found to cluster with the vaccine strain, whereas VP1 clustered with vvIBDV (A1aB2). Furthermore, the phylogenetic analysis of nVarIBDV (OR682618 and OR682619 isolates) in this study showed typical identity (100%) with nVarIBDV strains (A2d) circulating in China, such as IBD/SD/LY/CN/01/2020 (OM307063), and 99.5% similarity with ZD-2018-1 (MN485882). In addition, three major aa mutations were detected in both nVarIBDV isolates (OR682618 and OR682619) as D213N which is responsible for immune escape [65], and D279N, and A321V, which are markers of reduced pathogenicity and altered antigenicity in variant strains [66,67]. Therefore, this new genetic variant (A2d) is added to the IBDV genotypes recorded in Egypt, which include A1aB1, A1bB1, A1aB2, A2d, A3B2, A3B1, A7B3 and A2dB1b [19,24,25,27,64,68,69,70,71,72]. It is well documented that nVarIBDV suppresses the immune system, hence indicating the economic importance of this hidden virus in the low performance of the affected flocks in association with secondary infections such as LPAI-H9N2, mycoplasma, *Escherichia coli* and necrotic enteritis (*Clostridium perfringens*). Although the flocks studied were vaccinated with both recombinant and live vaccines, the emergence of nVarIBDV strains highlights the importance of vaccinating breeder chickens with inactivated vaccines containing this novel variant strain. The percentage of genetic similarity in VP2-HVR of nVarIBDV, A2d, isolated in 2023 and other 18 vaccine strains (4 from A1, 2 from A3, 12 from A9, genotypes) ranged from 86 to 90.4% (Figure 5), with the highest similarity to Faragher 52/70 (A1) (Vaxxitek^®^), the recombinant vaccine strain, which may be insufficient to protect against nVarIBDV. Therefore, the use of inactivated vaccines containing nVarIBDV would be necessary to use in breeder chickens in order to effectively protect their offspring during the first 2–3 weeks of life. Finally, there are fears of future reassortment of A2d with A3 IBDV strains, creating a new genotype with increased pathogenicity, as has occurred in China [73].

## 5. Conclusions

The current research documents the presence of nVarIBDV in Egypt through viral isolation and partial VP2 sequencing. Accordingly, further research is needed to investigate the epidemiological landscape of this novel genotype across Egypt and to assess the efficacy of current vaccine regimes against it. Inclusion of this variant strain in the inactivated infectious bursal disease (IBD) vaccines used in breeder hens prior to egg production, is essential to obtain maternal antibodies that will specifically protect their broiler chicks against the novel variant infectious bursal disease virus (nVarIBDV) during the first three weeks of life.

## Figures and Tables

**Table 1:** History of nVarIBD in Egyptian chicken flocks during 2023.

Flock No.	Locality	Type of Chickens	Total No.	Age (Days)	Types and Age of IBD Vaccination Used	Mortality Rate % during 5 Days of Infection	Sample Origin
1	New Valley	Commercial broiler	40,000	20	Recombinant (1 DO) and live IM (14 DO)	0.2	Bursa homogenate
2	New Valley		40,000	13		0.3	
3	Alexandria		12,000	18	Live IM (7 DO) and IMP (14 DO)	1	
4	Alexandria		14,000	21		0.5	
5	Beheira		10,000	17	ICX (1 DO) and live IM (14 DO)	1.2	
6	Beheira		9000	20		1.5	
7	Alexandria		15,000	18		0.4	
8	Beheira		14,000	21	Live IMP (14 DO)	2	
9	Beheira		10,000	26		2.1	
10	Alexandria		7000	16	Live IM (7 DO) and IMP (14 DO)	1	
11	Alexandria		20,000	18		2	
12	Beheira		9000	21		1.4	
13	Beheira		6000	22	Recombinant (1 DO) and live IM (14 DO)	1	
14	Beheira		7000	19		0.9	
15	Alexandria		10,000	25		0.6	
16	Beheira	Indigenous Balady Breed	6000	49		0.3	Bursa and spleen homogenate
17	Beheira		4500	35		2	
18	Beheira		6700	19	Live IM (7 DO) and IMP (14 DO)	1.5	

DO: days old; IM: intermediate; IMP: intermediate plus; ICX: immunocomplex.

## Data Availability

The datasets used during this study are available from the corresponding author on reasonable request.
